# *Eucheuma cottonii*–enriched legume-based green concentrate improves rumen protein utilization, methane mitigation, and lactation performance in Holstein Friesian dairy cows

**DOI:** 10.14202/vetworld.2026.2144-2159

**Published:** 2026-05-21

**Authors:** Renny Fatmyah Utamy, Ambo Ako, Zyahrul Ramadan, Jamila Mustabi, Mohammad Mijanur Rahman, Kannika Umpuch

**Affiliations:** 1 Department of Animal Production, Faculty of Animal Science, Hasanuddin University, Makassar, South Sulawesi, Indonesia; 2 Department of Animal Nutrition, Faculty of Animal Science, Hasanuddin University, Makassar, South Sulawesi, Indonesia; 3 Livestock Production Programme, Faculty of Sustainable Agriculture, Universiti Malaysia Sabah, Sandakan, Sabah, Malaysia; 4 Department of Agriculture, Faculty of Agricultural Technology, Valaya Alongkorn Rajabhat University under the Royal Patronage, Pathum Thani, Thailand

**Keywords:** dairy nutrition, *Eucheuma cottonii*, feed efficiency, green concentrate, methane mitigation, milk yield, rumen-protected protein, sustainability

## Abstract

**Background and Aim:**

Legume-based green concentrate (GC) is a promising, low-cost feeding strategy for dairy systems; however, its high rumen-degradable protein (RDP) fraction limits nitrogen utilization efficiency. This study evaluated the effect of *Eucheuma cottonii* enrichment as a rumen-protected agent on feed quality, rumen protein utilization, methane mitigation, and lactation performance in Holstein Friesian dairy cows.

**Materials and Methods:**

The experiment comprised integrated *in vitro* and *in vivo* phases. In the *in vitro* phase, GC was enriched with *E. cottonii* at 0%, 2.5%, 5%, 7.5%, and 10% and assessed for digestibility, rumen protein utilization (RDP and rumen-undegradable protein [RUP]), gas production, and fermentation characteristics. In the *in vivo* phase, eighteen postpartum cows were randomly assigned to three dietary treatments: commercial concentrate, GC, and GC enriched with 10% *E. cottonii* for 60 days. Parameters included milk yield, dry matter intake, feed conversion efficiency (FCE), milk quality, and economic returns.

**Results:**

*E. cottonii* enrichment significantly improved feed quality and rumen protein utilization. A marked reduction in RDP and a concurrent increase in RUP were observed (p < 0.01), indicating enhanced protein bypass. Digestibility indices, including *in vitro* dry matter, organic matter, crude protein, and gross energy digestibility, were significantly increased (p ≤ 0.002). Methane production and total gas output were reduced (p ≤ 0.01), reflecting improved rumen fermentation efficiency. In the *in vivo* trial, cows fed 10% enriched GC exhibited higher milk yield (p = 0.01), improved FCE (p = 0.02), and enhanced milk fat and protein contents (p < 0.01). Economic analysis demonstrated reduced feed costs and increased daily profit. The functional mechanism is attributed to kappa-carrageenan in *E. cottonii*, which forms a protective matrix limiting microbial proteolysis.

**Conclusion:**

Enrichment of GC with *E. cottonii* effectively optimizes rumen protein utilization, enhances digestibility, reduces methane emissions, and improves lactation performance and farm profitability. This strategy represents a sustainable, cost-effective innovation for improving dairy productivity and environmental efficiency.

## INTRODUCTION

Average milk yield in Indonesia remains low at approximately 9–10 L/cow/day [1]. Beyond environmental constraints associated with tropical climates [2], milk production is strongly determined by feed quality. Nutritional inadequacies—particularly protein and energy—depress milk yield [3], whereas mineral imbalances (e.g., calcium) may precipitate hypocalcemia manifested as muscular stiffness or paresis [4]. Furthermore, early lactation is frequently characterized by negative energy balance (NEB), which heightens metabolic stress and compromises performance [5].

Concentrate feeds commonly offered to dairy cows are commercial formulations that are relatively costly and sporadically available. Consequently, farmers often limit concentrate provision, with downstream impacts on milk output. Developing locally available, accessible, and cost-effective alternatives is therefore a pressing need. Legume-based green concentrate (GC) represents a promising option because its protein sources derive from legumes such as *Indigofera zollingeriana* (crude protein, ~26%) and *Gliricidia sepium* (crude protein, ~24%), offering potential substitution for soybean meal, coconut meal, and fish meal, ingredients that are frequently imported and difficult to procure [6]. Reported benefits of GC include improved milk production [7], enhanced calf growth [8], reduced feed costs [5], and mitigation of NEB [6].

However, a key limitation of GC is its high proportion of rumen-degradable protein (RDP), which predisposes nitrogen losses in the rumen and constrains the post-ruminal supply of amino acids [7]. To address this limitation, protein protection technologies—namely rumen-protected agents (RPA), have been developed to increase rumen-undegradable protein (RUP) and improve intestinal amino acid delivery [9]. Among encapsulating materials, polysaccharides such as chitosan can form ionic gel networks in the rumen, thereby retarding proteolysis and improving nutrient utilization [10]. Chitosan has also been shown to enhance daily weight gain and feed efficiency in broilers [11].

Despite its efficacy, chitosan extraction from crab shells and other sources is technically demanding and costly for smallholder farmers. Kappa-carrageenan, a sulfated polysaccharide found predominantly in the seaweed *Eucheuma cottonii* (often exceeding 60%), offers a practical and cost-effective alternative. Carrageenan can function as a microencapsulant and RPA and has been applied in dairy systems [12, 13].

To date, the enrichment of GC with *E. cottonii* has not been systematically investigated in dairy cows. There is a clear lack of integrated studies linking this approach to comprehensive feed quality parameters, including proximate composition and rumen protein utilization, as well as fermentation characteristics, gas production, and nutrient digestibility. Moreover, limited evidence exists connecting these nutritional parameters with *in vivo* outcomes such as milk yield, feed conversion efficiency (FCE), body condition score (BCS), milk quality, and farm-level economic returns. Previous research has largely focused on isolated aspects, such as *in vitro* digestibility or production performance, without integrating metabolic, nutritional, and economic dimensions into a unified framework. Additionally, comparative evaluation of different inclusion levels of *E. cottonii* as an RPA in GC remains insufficient, limiting the ability to optimize feeding strategies for practical field application. Although earlier *in vitro* studies have reported methane mitigation properties of *E. cottonii*, its application as a microencapsulation-based rumen-protective technology within GC has not been fully explored, particularly under conditions relevant to smallholder dairy production systems.

Therefore, this study aimed to evaluate the effect of legume-based GC enriched with *E. cottonii* as an RPA to reduce RDP, increase RUP, and improve the productive performance of Holstein Friesian (HF) dairy cows. Specifically, the study assessed the impacts on feed quality and rumen performance (proximate composition, RDP/RUP, gas production, fermentation characteristics, and *in vitro* digestibility including *in vitro* dry matter digestibility (IVDMD), *in vitro* organic matter digestibility (IVOMD), *in vitro* crude protein digestibility (IVCPD), and *in vitro* gross energy digestibility (IVGED)), productive performance (milk yield, FCE, and BCS), milk quality (fat and protein contents, whey and curd yield), and economic indicators (daily feed cost and daily profit) compared with unfortified GC and commercial concentrate (CON).

## MATERIALS AND METHODS

### Ethical approval

All experimental procedures were conducted in accordance with institutional animal welfare guidelines. Ethical approval was obtained from the Ethics Committee on the Use of Research and Learning Animals, Faculty of Animal Science, Hasanuddin University, Indonesia (Approval No. 030/UN4.12/EC/VIII/2025). The dairy cows used in this study were clinically healthy and free from mastitis. Routine mastitis monitoring was conducted every 14 days at the research site, and post-milking teat dipping was consistently implemented as a preventive measure. Prior to animal allocation, a screening process was undertaken to ensure homogeneity in physiological and production characteristics. Randomization of experimental animals was then performed manually using a lottery procedure to ensure unbiased group assignment.

### Study period and location

The experiment was conducted from August 8 to October 7, 2025 in Lebang Village, Cendana District, Enrekang Regency, South Sulawesi, Indonesia. The research location was 3°29′12.12″ South Latitude and 119°51′40.319″ East Longitude, at an altitude of 47 m above sea level (map coordinates). The study was done under real farm conditions. The average temperature in the cage ranged from 24°C (morning) to 34°C (afternoon), relative humidity was 85% (morning) and 52% (afternoon), and the cage received approximately 13 h of natural light (calculated during the research period). However, sample testing was conducted in the laboratory as an indicator of inter-treatment assessment after implementation in dairy cows to ensure the accuracy and validity of livestock performance evaluation results. The milk quality was analyzed at the Dairy Production Laboratory, Faculty of Animal Science, Hasanuddin University, Makassar. Quality of the diet was analyzed at the Dairy Animal Nutrition Laboratory, IPB University, Bogor, West Java.

### Study design

This study was divided into two stages, including *in vitro* design and *in vivo* design, as follows:

***In vitro* phase**: The formulated GC was subsequently enriched with an RPA in the form of *E. cottonii* at varying inclusion levels. The treatments consisted of: GC without enrichment (GC); GC enriched with 2.5% *E. cottonii* meal (GCC1); GC enriched with 5% (GCC2); GC enriched with 7.5% (GCC3); and GC enriched with 10% (GCC4).

All treatments were subjected to *in vitro* evaluation, which included determination of IVDMD, IVOMD, IVCPD, IVGED, rumen protein utilization (RDP and RUP), gas production, and rumen fermentation characteristics (volatile fatty acids [VFAs]).

***In vivo* phase and animal selection**: Eighteen multiparous HF dairy cows (aged 4–5 years; average body weight ≈ 500 kg) in early lactation (20–40 days postpartum) with an initial milk yield of 8–9 kg/day were enrolled in the study. Dairy cows were randomly allocated to three dietary treatments using a completely randomized design (n = 6 per group). This sample size is considered adequate to support the conclusions of the study. The six replicates used represent a cohort that is sufficiently representative of the target population. All cows included in the experiment were standardized according to the predetermined selection criteria, ensuring homogeneity prior to treatment allocation.

The treatments consisted of CON, legume-based GC without enrichment (GC), and GC enriched with 10% *E. cottonii* meal (GCC4). The inclusion level of 10% was selected based on prior *in vitro* evaluations, which indicated superior performance in terms of feed quality and rumen performance. Randomization was conducted using a manual lottery system to ensure equal allocation probability, thereby enhancing the validity and reliability of the experimental design.

### Diet formulation and feeding management

All diets were formulated to comply with national standards for lactating dairy cows concentrates, ensuring a minimum of 18% crude protein (CP) and 70% total digestible nutrients (Table 1).

**Table 1:** Composition of diets under treatments.

Feedstuffs	CON	GC
Soybean meal	24	13
Rice bran	38	36
Molasses	2	2
Corn Meal	36	29
Indigofera leaves meal	0	5
Gliricidia leaves meal	0	15
Total	100	100

CON = Commercial concentrate, GC = Green concentrate.

**Figure 1:**
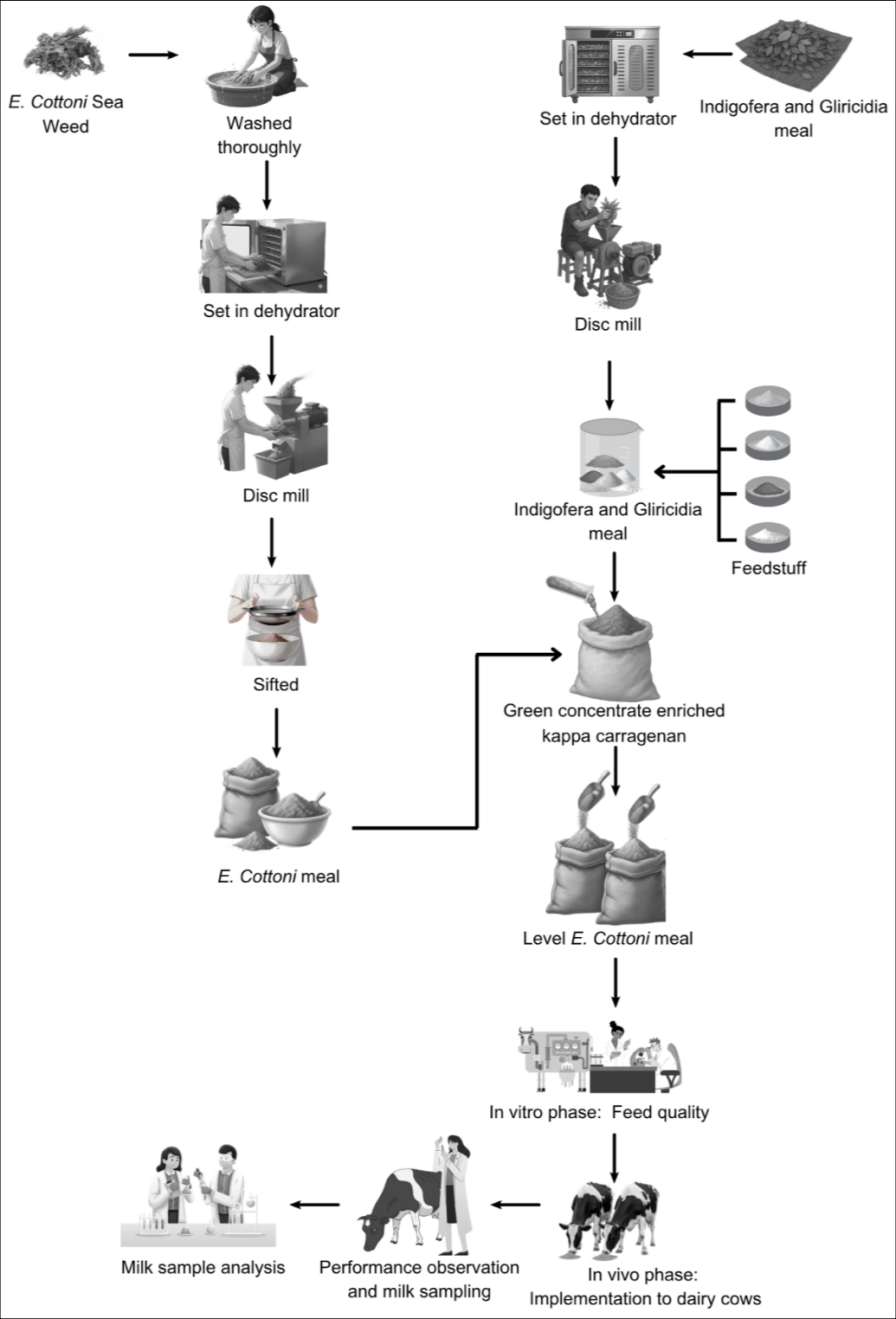
The diagram summarizes the experimental workflow, including diet formulation, *E. cottonii* enrichment, *in vitro* evaluation, *in vivo* feeding trial, rumen fermentation assessment, performance evaluation, milk quality assessment, and economic analysis.

Legume components, Gliricidia and Indigofera leaves, were harvested, dehydrated at 70°C for 7 h, and subsequently ground into meal using a disc mill. The same procedure was applied to *E. cottonii* seaweed to produce seaweed meal rich in kappa-carrageenan (Figure 1), nutritional values of *E. cottonii* refer to Table 2 [13, 14].

**Table 2:** Nutritional value of commercial concentrate and *Eucheuma cottonii*.

Nutritional composition (%)	Commercial concentrate	*E. cottonii*
CP	18.79	6.31
CF	5.93	0.09
CFi	5.95	7.86
Ash	4.33	13.70
Kappa-carrageenan	–	60

CP = Crude protein, CF = Crude fat, CFi = Crude fiber, Nutritional values of *E. cottonii* were adopted from Natsir *et al*. [14], kappa-carrageenan value was adopted from Anggraini *et al*. [13].

Seaweed *E. cottonii* is cultivated on the west coast of South Sulawesi, specifically in Takalar Regency. It is harvested at 45 days old, cleaned of contaminants such as small shells and other debris. It is then rinsed three times with fresh water and drained. After that, it is placed in a dehydrator at 70°C for 24 h. Once *E. cottonii* is dry, it is then disc milled into 1 mm meal that is ready for use in GC.

The commercial concentrate (Sipatuo®, CV Sipatuo Farm, Enrekang, Indonesia) used as a reference treatment was sourced from CV Sipatuo Farm; nutritional values refer to Table 2, a widely utilized brand among local dairy producers.

Cows were fed at 3% of body weight on a dry matter basis, with a concentrate-to-forage ratio of 30:70, using elephant grass (*Pennisetum purpureum*) as the forage component. The feeding trial lasted for 60 days postpartum, following a 7-day adaptation period. Dairy cows were housed in individual pens and offered feed three times daily at 06:00 am, 11:00 am, and 04:00 pm.

### Chemical composition analysis of the diet

Feed samples were analyzed for proximate composition following Association of Official Analytical Chemists (AOAC) protocols: CP; AOAC 976.05), crude fat (CF; AOAC 989.04), crude fiber (CFi; AOAC 962.09), nitrogen-free extract (NFE; AOAC 2003.05), and ash content (AOAC 942.05). Gross energy (GE) was determined using a bomb calorimeter (Model XRY-1A+, China, calibrated with benzoic acid standard), which measures calorific value based on the heat released during complete combustion of the sample in an oxygen-rich environment [15].

### *In vitro* rumen fermentation and digestibility analysis

Rumen fluid was obtained from HF dairy cows kept in the field laboratory. The cows used had been fistulated to facilitate rumen fluid collection. Rumen fluid collection was carried out in the morning to ensure that the microenvironment was more representative. Dairy cows were fed a 70:30 forage:concentrate diet and strained through cheesecloth under CO_2_ flushing.

VFAs are produced during microbial fermentation of carbohydrates and proteins in the rumen. Major VFAs include acetate, propionate, isopropionate, butyrate, isobutyrate, valerate, and isovalerate. VFAs concentrations were analyzed using gas chromatography, based on differential absorption and partitioning across stationary and mobile phases. Separation resulted in distinct peaks on the chromatogram. Sample concentrations were quantified by comparing peak areas to those of known reference standards.

*In vitro* digestibility was assessed following preparation of McDougall’s buffer and fresh rumen fluid. Each fermentor tube was filled with 0.5 g of sample and 40 mL of McDougall’s buffer. The tubes were incubated in a shaker water bath at 39°C, and 10 mL of rumen fluid was added. A stream of CO_2_ was applied for 30 s, and pH was adjusted to 6.5–6.9. Tubes were sealed with rubber stoppers and incubated anaerobically for 48 h. Post-incubation, 2–3 drops of HgCl_2_ were added to terminate microbial activity. The contents were centrifuged at 5,000 rpm for 15 min to separate supernatant and residue. The precipitate was incubated in 50 mL of 0.2% pepsin-HCl solution for 48 h at 39°C. Digested residues were filtered under vacuum using pre-weighed Whatman No. 41 filter paper. The filter paper containing residue was oven-dried at 105°C for 24 h. Dried samples were weighed to determine dry matter content. Samples were ashed at 450–600°C for 6 h to calculate organic matter content. Blank tubes (without feed) were included to correct for microbial residue in CP and GE analysis.

Digestibility was calculated using the Tilley and Terry two-stage technique [16]:

%IV digestibility = (initial weight (g) − (residual weight (g) − blank correction)) / (initial weight (g)) × 100%

The RDP and RUP levels, as well as rumen fluid characteristics *in vitro* were measured using the Tilley and Terry method [15]. The same method as for *in vitro* digestibility. Then centrifugation was carried out to separate the supernatant and residue at 1,006 × *g* at 40°C. The residue that has been filtered with Whatman No. 41 filter paper was used to determine the digestibility and content of RDP–RUP following the proximate analysis method.

The gas production was measured every 2 h using a 10 mL syringe with a 0.1 mm needle injected into the rubber part of the sealed bottle during 24 h *in vitro* incubation and collected into the vacuumed and sealed 100 mL bottle. The 24-h recordings of gas production from each bottle were summarized for the total gas production, while the gas kinetics data were analyzed using an exponential model [17]. Moreover, about 5 mL of the total gas was taken and collected in a 5 mL Vacutainer for methane concentration measurement. The methane analysis used the Shimadzu GC-8A with a flame ionization detector following Haryati *et al*. [18].

### Animal performance evaluation

Milk yield and dry matter (DM) intake were monitored over a 60-day period. Dry matter intake (DMI) is determined by subtracting the daily feed residue from the amount of feed offered (DM). The feed residue is weighed each day to provide the actual DMI value. FCE was calculated as the ratio of milk yield to DM intake (milk yield ÷ DM intake).

BCS was assessed on days 0, 30, and 60 of the trial. BCS was used as an indicator of cow health and was determined through visual inspection and palpation. BCS was evaluated following Edmonson *et al*. [19] based on eight anatomical landmarks, including the spinous and transverse processes, rumen fill, tuber coxae, tuber ischii, and the area between the hook and tail head. Cows were observed on a flat surface from multiple angles (side, front, and rear) at a distance of 2 m to ensure accurate BCS scoring. BCS was scored on a 1–5 scale: 1 = emaciated, 2 = thin, 3 = moderate, 4 = fat, and 5 = obese. Intermediate scores (e.g., 0.25, 0.5, 0.75) were used for more precise estimation.

### Economic analysis

Feed input costs (USD/kg DM) were calculated using prevailing market prices for each concentrate component. The GC cost was determined based on the current market price of its raw ingredients. Although forage was home-grown, costs were estimated based on fertilizer, labor, and transportation expenses.

Daily feed cost (USD/head/day) was computed by multiplying feed price (USD/kg DM) by feed intake (kg DM/head/day). Income from daily milk yield (USD/head/day) was calculated as milk yield (kg/day) × milk price (USD/kg). Daily profit (USD/head/day) was obtained by subtracting daily feed cost from daily milk income:

Daily profit = milk income − feed cost

Daily profit = (milk yield × milk price) − (DMI concentrate × concentrate price + DMI forage × forage price)

### Milk quality assessment

On day 60, composite milk samples were collected from each cow for laboratory analysis. Approximately 100 mL of milk was obtained per animal, immediately placed in sterile containers, and stored in a cooler box to maintain sample integrity during transport to the laboratory. During transportation, the cooler box temperature remained stable at 4°C for a duration of 5 h.

Subsequent analyses included crude fat, protein, lactose, solids-not-fat (SNF), inorganic salt, and density, assessed using an automatic milk analyzer (Infitek MA-H3, China). The analyzer was calibrated daily using standard milk samples. Milk samples (50 mL) were homogenized at 3,000 rpm for 30 s and analyzed under controlled laboratory conditions (18–24°C). Two repetitions were carried out on the same milk sample, and the results used were the average of these tests.

Milk curd and whey percentages were measured by heating 1 kg of fresh milk using the low-temperature, long-time method. Then, 4 mL of papain enzyme from papaya latex solution was added. After separation, curd and whey were weighed and calculated following Sutomo *et al*. [20]:

%Curd = (curd weight) / (milk initial weight (g)) × 100%

%Whey = (whey weight) / (milk initial weight (g)) × 100%

### Statistical analysis

Data were analyzed using one-way analysis of variance in SPSS version 27.0 (IBM Corp., Armonk, NY, USA). The model used was:

Yij = μ + τi + εij

Where Yij is the observed value, μ is the overall mean, τi is the treatment effect, and εij is the residual error. Where significant differences were found, Duncan’s multiple range test was used for post hoc comparison (p < 0.05). Data normality was tested using the Shapiro–Wilk test, and homogeneity was assessed using Levene’s test. Pearson correlation analysis was conducted to evaluate the relationship between the inclusion level of *E. cottonii* (%) and feed quality.

## RESULTS

### Chemical analysis, rumen protein, gas production, rumen fermentation, and nutrient digestibility of the diets

Statistical analysis (Table 3) revealed that enrichment of GC with microencapsulated *E. cottonii* significantly affected several proximate components: organic matter (OM) (p < 0.00), crude fat (CF) (p = 0.02), and crude fiber (CFi) (p < 0.00). No significant differences were observed for DM (p = 0.27) and CP (p = 0.15).

**Table 3:** Chemical analysis, rumen protein, gas production, rumen fermentation, and nutrient digestibility of GC fortified with *Eucheuma cottoni.*

Parameters	GC	GCC1	GCC2	GCC3	GCC4	SEM	p-value
Chemical analysis (%)							
CP	20.10 ± 0.82	19.13 ± 0.60	18.87 ± 0.72	18.96 ± 0.35	18.65 ± 0.72	0.19	0.152
CF	5.23 ± 0.12ᵇ	4.93 ± 0.33ᵇ	4.47 ± 0.19ᵃᵇ	4.42 ± 0.49ᵃᵇ	3.81 ± 0.71ᵃ	0.16	0.020
CFi	5.30 ± 0.47ᵃ	6.49 ± 0.25ᵇ	6.45 ± 0.19ᵇ	6.51 ± 0.34ᵇ	6.68 ± 0.31ᵇ	0.15	0.003
Rumen protein utilization (%)							
RDP	72.18 ± 1.25ᶜ	69.53 ± 0.52ᵇ	69.40 ± 0.68ᵇ	66.70 ± 1.15ᵃ	65.24 ± 1.17ᵃ	0.68	0.000
RUP	59.56 ± 0.85ᵃ	60.43 ± 1.18ᵃᵇ	60.57 ± 1.12ᵃᵇ	62.42 ± 1.40ᵇᶜ	64.17 ± 1.09ᶜ	0.50	0.004
Gas production							
Methane gas (% of total gas)	11.09 ± 0.13ᶜ	10.86 ± 0.62ᵇᶜ	10.24 ± 0.46ᵃᵇ	9.85 ± 0.31ᵃ	9.76 ± 0.41ᵃ	0.16	0.011
Total gas (mL/g)	57.00 ± 2.00ᶜ	56.66 ± 1.15ᶜ	55.00 ± 3.46ᵇᶜ	52.66 ± 1.15ᵃᵇ	50.00 ± 1.00ᵃ	0.82	0.007
Rumen fermentation (mmol/L)							
Acetate	50.57 ± 10.69ᵃ	52.51 ± 21.65ᵃ	53.68 ± 20.78ᵃ	32.07 ± 10.95ᵃ	83.09 ± 12.04ᵇ	5.59	0.036
Propionate	15.21 ± 4.19ᵃ	28.27 ± 6.27ᵇ	18.45 ± 4.19ᵃ	11.68 ± 3.61ᵃ	38.28 ± 3.41ᶜ	2.76	0.000
Isobutyrate	0.43 ± 0.00ᵃ	0.76 ± 0.14ᵃ	0.57 ± 0.14ᵃ	0.67 ± 0.37ᵃ	1.26 ± 0.24ᵇ	0.89	0.009
Butyrate	4.18 ± 0.03ᵃ	7.28 ± 1.15ᵇ	5.28 ± 0.98ᵃᵇ	6.17 ± 1.91ᵃᵇ	10.16 ± 1.52ᶜ	0.61	0.002
Isovalerate	0.50 ± 0.01ᵃ	0.88 ± 0.09ᵃ	0.66 ± 0.16ᵃ	0.81 ± 0.40ᵃ	1.47 ± 0.32ᵇ	0.10	0.006
Valerate	0.31 ± 0.01ᵃ	0.55 ± 0.04ᵇ	0.41 ± 0.10ᵃᵇ	0.44 ± 0.15ᵃᵇ	1.07 ± 0.16ᶜ	0.07	0.000
VFA	88.30 ± 11.75ᵃ	124.92 ± 6.05ᵇ	136.37 ± 4.47ᶜ	139.02 ± 4.51ᶜᵈ	146.38 ± 4.85ᵈ	4.37	0.000
*In vitro* digestibility (%)							
IVDMD	69.65 ± 0.24ᵃ	72.90 ± 0.55ᵇ	74.53 ± 0.25ᶜ	77.60 ± 0.08ᵈ	78.77 ± 0.65ᵉ	0.88	0.000
IVOMD	68.58 ± 0.22ᵃ	72.12 ± 0.57ᵇ	74.07 ± 0.25ᶜ	77.28 ± 0.15ᵈ	78.12 ± 0.70ᵉ	0.93	0.000
IVCPD	81.96 ± 0.15ᵃ	83.32 ± 0.24ᵇ	84.54 ± 0.03ᶜ	85.29 ± 0.16ᵈ	88.03 ± 0.62ᵉ	0.54	0.000
IVGED	59.25 ± 0.33ᵃ	65.91 ± 0.69ᵇ	68.68 ± 0.32ᶜ	69.52 ± 0.11ᶜ	74.40 ± 0.90ᵈ	1.33	0.000

Different superscripts in the same row indicate significant differences (p < 0.05), SEM = Standard error of the means, GC = Green concentrate, GCC1 = GC fortified with 2.5% *E. cottonii*, GCC2 = GC fortified with 5% *E. cottonii*, GCC3 = GC fortified with 7.5% *E. cottonii*, GCC4 = GC fortified with 10% *E. cottonii*, CP = Crude protein, CF = Crude fat, CFi = Crude fiber, RDP = Rumen-degradable protein, RUP = Rumen-undegradable protein, VFA = Volatile fatty acid, IVDMD = *In vitro* dry matter digestibility, IVOMD = *In vitro* organic matter digestibility, IVCPD = *In vitro* crude protein digestibility, IVGED = *In vitro* gross energy digestibility.

**Figure 2:**
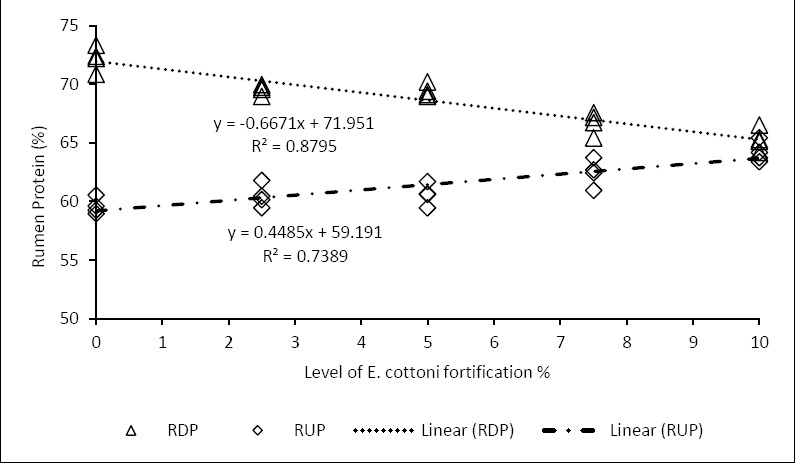
Correlation between *Eucheuma cottonii* fortification level and rumen protein utilization. Increasing *E. cottonii* fortification decreased rumen-degradable protein (RDP) and increased rumen-undegradable protein (RUP), indicating improved protein protection and greater bypass protein availability.

Rumen performance parameters were markedly influenced by dietary treatment. Increasing levels of *E. cottonii* enrichment significantly reduced RDP (p < 0.00) while increasing RUP (p < 0.00) (Table 3). The level of *E. cottonii* fortification (%) had a strong correlation with RDP (R² = 0.87) and RUP (R² = 0.73) (Figure 2).

**Figure 3:**
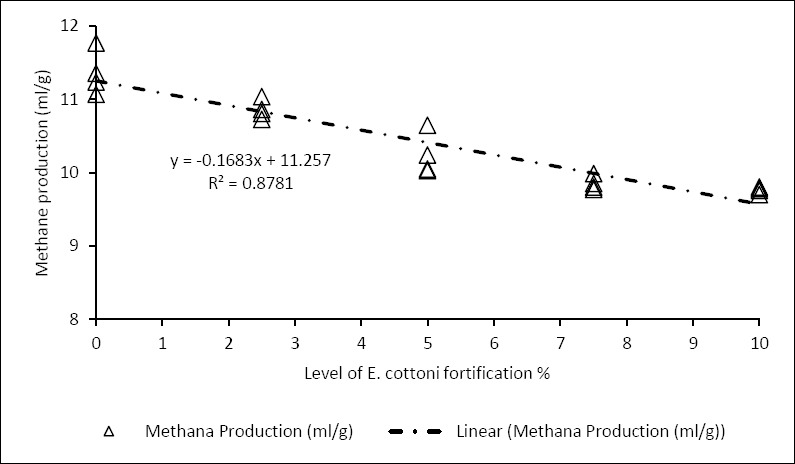
Correlation between *Eucheuma cottonii* fortification level and methane production. Increasing *E. cottonii* fortification reduced methane production, indicating improved rumen fermentation efficiency and methane mitigation potential.

Furthermore, enrichment mitigated methane accumulation, as evidenced by significant reductions in methane production (p = 0.01) and total gas output (p < 0.00) (Table 3). There was a strong correlation (R² = 0.87) between the level of *E. cottonii* fortification (%) and methane production (Figure 3).

**Figure 4:**
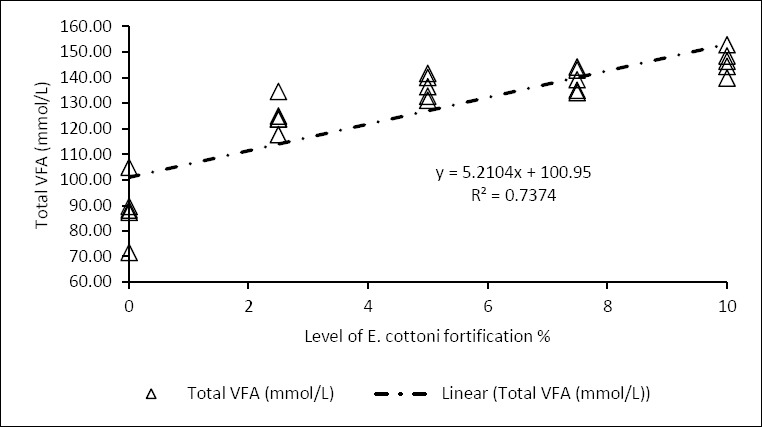
Correlation between *Eucheuma cottonii* fortification level and total volatile fatty acid concentration. Increasing *E. cottonii* fortification increased total volatile fatty acid (VFA) concentration, indicating enhanced ruminal fermentation and improved nutrient utilization.

Rumen protein utilization were also significantly altered. Enrichment with *E. cottonii* increased concentrations of individual VFA, including acetate (p = 0.03), propionate (p < 0.00), butyrate (p < 0.00), isobutyrate (p < 0.00), valerate (p < 0.00), and isovalerate (p < 0.00), as well as total VFAs (p < 0.00) (Table 3). There was a strong correlation (R² = 0.73) between the level of *E. cottonii* fortification (%) and total VFA (Figure 4).

**Figure 5:**
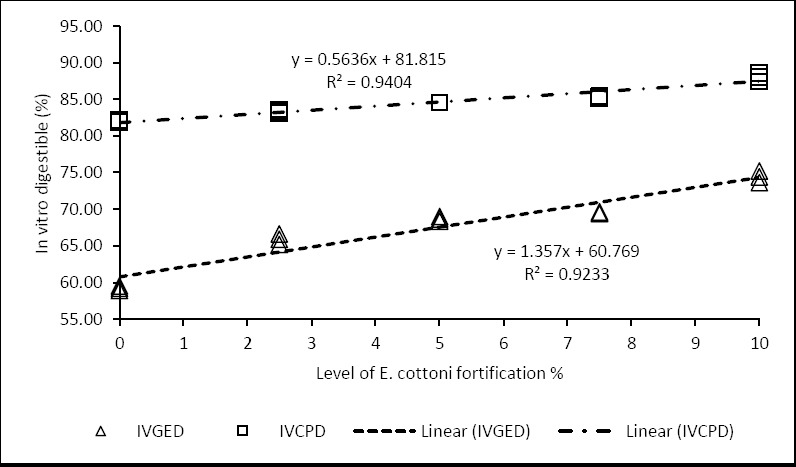
Correlation between *Eucheuma cottonii* fortification level and *in vitro* digestibility. Increasing *E.cottonii* fortification improved *in vitro* digestibility, supporting enhanced nutrient availability in green concentrate enriched with *E. cottonii*. IVGED = *In vitro* gross energy digestibility, IVCPD = *In vitro* crude protein digestibility.

*In vitro* digestibility improved significantly with enrichment. Higher values were recorded for IVDMD (p < 0.00), IVOMD (p < 0.00), IVCPD (p < 0.00), and IVGED (p < 0.00) compared to the unenriched control (Table 3). The level of *E. cottonii* fortification (%) had a strong correlation with IVGED (R² = 0.92) and IVCPD (R² = 0.94) (Figure 5).

### Performance, economic benefit, and milk quality of HF dairy cows fed GC fortified with *E. cottonii*

Based on Table 4, GC enriched with 10% *E. cottonii* (GCC4) significantly improved milk yield (p = 0.01) and FCE (p = 0.02), while no significant differences were observed for DMI (p = 0.49) and BCS (p = 0.17). Economic analysis indicated that GCC4 diets significantly reduced daily feed costs (p < 0.00), increased daily profit (p = 0.01), and enhanced income from milk yield (p = 0.04). Milk quality was positively influenced by GCC4 enrichment, with significant increases in fat content (p < 0.00), protein content (p < 0.00), and whey and curd yield (p < 0.00). However, lactose (p = 0.43), SNF (p = 0.11), and milk density (p = 0.46) remained unaffected.

**Table 4:** Performance, milk quality, and economic benefits of Holstein–Friesian dairy cows fed green concentrate fortified with *Eucheuma cottonii*.

Parameters	CON	GC	GCC4	SEM	p-value
Production performance					
Milk yield (kg/day)	8.67 ± 1.03ᵃ	9.36 ± 0.49ᵃᵇ	10.05 ± 0.24ᵇ	0.20	0.011
DMI (kg/day)	10.63 ± 0.16	10.81 ± 0.38	10.81 ± 0.32	0.07	0.496
FCE	0.81 ± 0.09ᵃ	0.86 ± 0.03ᵃᵇ	0.92 ± 0.04ᵇ	0.01	0.022
BCS	2.50 ± 0.15	2.45 ± 0.24	2.65 ± 0.12	0.04	0.177
Economic benefit					
Feed input (USD/kg DM)					
Concentrate	0.45	0.30	0.33	–	–
Forage	0.15	0.15	0.15	–	–
Daily feed cost (USD/day/head)	2.81 ± 0.02ᶜ	2.29 ± 0.06ᵃ	2.43 ± 0.05ᵇ	0.22	0.000
Income from daily production (USD/day/head)	9.97 ± 1.33ᵃ	10.76 ± 0.64ᵃᵇ	11.56 ± 0.31ᵇ	0.27	0.044
Daily profit (USD/day/head)	7.16 ± 1.31ᵃ	8.74 ± 0.61ᵇ	9.13 ± 0.33ᵇ	0.30	0.011
Milk quality					
Fat (%)	3.85 ± 0.36ᵃ	4.03 ± 0.29ᵃ	5.90 ± 1.20ᵇ	0.27	0.000
Protein (%)	3.12 ± 0.03ᵃ	3.13 ± 0.05ᵃ	3.34 ± 0.08ᵇ	0.02	0.000
Lactose (%)	4.88 ± 0.22	4.82 ± 0.20	4.90 ± 0.18	0.04	0.432
SNF (%)	8.75 ± 0.28	8.78 ± 0.38	8.96 ± 0.32	0.07	0.115
Density (kg/m³)	1029.91 ± 1.07	1029.88 ± 1.32	1030.12 ± 0.79	0.33	0.466
Whey (g/1000 g)	753.67 ± 16.17ᵇ	708.37 ± 14.84ᵃ	710.93 ± 26.19ᵃ	6.66	0.002
Curd (g/1000 g)	246.32 ± 16.17ᵃ	291.63 ± 14.84ᵇ	289.06 ± 26.19ᵇ	6.66	0.002

Different superscripts in the same row indicate significant differences (p < 0.05), SEM = Standard error of the means, HF = Holstein Friesian, NEB = Negative energy balance, CON = Commercial concentrate, GC = Green concentrate, GCC4 = GC fortified with 10% *E. cottonii*, DMI = Dry matter intake, FCE = Feed conversion efficiency, BCS = Body condition score, SNF = Solids-not-fat, DM = Dry matter.

## DISCUSSION

### Chemical composition of diet

Green concentrates, both enriched and unenriched with *E. cottonii*, exhibited chemical profiles consistent with National Research Council recommendations [21] (Table 3). Increasing levels of *E. cottonii* enrichment resulted in a significant reduction in CF (p = 0.020) and a non-significant decrease in CP (p = 0.152). This trend can be attributed to the inherently low CF and CP content of *E. cottonii*, which, when incorporated into the concentrate, proportionally reduces these components [22].

The decline in fat content may also be explained by the hydrophilic nature of seaweed meal, which preferentially binds water rather than fat. Typically, fat molecules are associated with the positive poles of proteins; however, the addition of seaweed meal shifts protein binding affinity toward water, thereby reducing its capacity to bind fat [23]. Conversely, CFi content increased with higher enrichment levels, reflecting the substantial fiber fraction of *E. cottonii*, primarily composed of kappa-carrageenan [22, 23].

Overall, fortification up to 10% *E. cottonii* maintained proximate composition (CP, CF, and CFi) within NRC standards [21], indicating that such inclusion levels are nutritionally acceptable for lactating dairy cows.

### Rumen protein characteristics

The RDP values exhibited a clear decreasing trend with increasing levels of encapsulated *E. cottonii* enrichment (Table 3), with a strong negative correlation (R² = 0.87) as shown in Figure 2. The control group (CON) recorded the highest RDP value, while the lowest was observed in GCC4 (10% enriched). This reduction strongly suggests that kappa-carrageenan from *E. cottonii* encapsulation effectively protects protein from rapid microbial degradation in the rumen.

The underlying mechanism is attributed to the presence of kappa-carrageenan from *E. cottonii*, a sulfated polysaccharide, which forms ionic gel matrixes under ruminal conditions. These matrixes create a physical barrier that limits microbial proteolysis, thereby reducing RDP and improving nitrogen utilization efficiency [9]. The recommended RDP threshold for optimal dairy cow performance is < 65%, and synchronizing RDP with fermentable carbohydrates (NFC) is essential to maximize microbial protein synthesis and prevent nitrogen losses [24].

Without RPA, excess RDP can lead to elevated ammonia (NH_3_) concentrations, which are absorbed into the bloodstream, converted to urea in the liver, and excreted in urine. Subsequent hydrolysis of urinary urea releases NH_3_ into the environment, contributing to nitrogen pollution [25]. Thus, the inclusion of kappa-carrageenan from *E. cottonii* as an RPA aligns with strategies such as tannin supplementation, which also reduces RDP [26]. The effects of *E. cottonii* enrichment mirror those of chitosan and tannins, both known for their ability to protect proteins from microbial degradation in the rumen [11]. Unlike chitosan, which requires costly extraction processes, kappa-carrageenan is abundant in seaweed and can be incorporated directly into feed, making it a practical and cost-effective alternative.

Beyond its role in protein protection, kappa-carrageenan possesses unique functional properties. Its gel-forming ability has been widely utilized in food systems to protect sensitive nutrients from degradation in harsh environments, including the rumen [27, 28]. Furthermore, kappa-carrageenan from *E. cottonii* exhibits bioactive properties, such as mitigating inflammatory responses by inhibiting Nucleotide-binding Oligomerization Domain-containing protein 2 (NOD2) receptor signaling and downstream pathway activation [29]. Polysaccharides derived from macro- and microalgae, including carrageenan, also demonstrate antimicrobial activity against pathogenic bacteria. One proposed mechanism involves disruption of microbial cell walls; kappa-carrageenan from *E. cottonii* binds to and destabilizes the outer membrane, causing leakage of cellular components and eventual bacterial cell death [30]. These multifunctional attributes underscore the potential of *E. cottonii* not only as an RPA but also as a compound contributing to improved gut health and feed efficiency.

Overall, the observed decrease in RDP and increase in RUP associated with higher fortification levels confirm the effectiveness of *E. cottonii* as an RPA in modulating rumen protein dynamics. By reducing nitrogen losses and enhancing amino acid delivery to the small intestine, this strategy supports improved milk yield, feed efficiency, and environmental sustainability in dairy systems.

The RUP refers to the fraction of dietary protein that escapes microbial degradation in the rumen and flows directly to the abomasum and small intestine, where it undergoes enzymatic digestion and absorption. Approximately 80% of RUP is absorbed as amino acids in the small intestine, making it a critical source of high-quality amino acids for highly productive ruminants. These amino acids are essential for supporting milk protein synthesis, metabolic functions, and overall lactation performance. Consequently, diets for high-yielding dairy cows must contain an adequate proportion of RUP to meet post-ruminal amino acid requirements [26].

In the present study, RUP values increased consistently with higher inclusion levels of encapsulated *E. cottonii* enrichment, with the control group (CON) exhibiting the lowest RUP, and GCC4 (10% enriched) achieving the highest. This trend was statistically significant (p = 0.004) and demonstrated a positive correlation (R² = 0.73), supporting the hypothesis that *E. cottonii* as an RPA enhances bypass protein availability and improves protein delivery to the lower digestive tract. These findings align with previous research emphasizing the role of encapsulation technologies in modulating nutrient release and protecting proteins from ruminal degradation [31].

The mechanism underlying this improvement is based on the content of *E. cottonii*, i.e., kappa-carrageenan, a sulfated polysaccharide capable of forming an ionic gel matrix that acts as a physical barrier against microbial proteolysis. This controlled-release system ensures that a greater proportion of dietary protein bypasses ruminal fermentation and becomes available for enzymatic digestion in the small intestine. Similar effects have been reported with tannins, which naturally occur in legumes used in GC formulations and are known to reduce ruminal protein degradation while increasing RUP [32]. By combining the inherent tannin content of legumes with *E. cottonii*-enriched concentrate, the GC provides a dual mechanism for protein protection, thereby optimizing nitrogen utilization efficiency and supporting improved lactation performance.

Overall, the observed increase in RUP with higher enrichment levels demonstrates the practical and nutritional significance of incorporating *E. cottonii*-based encapsulation into dairy cow diets. This strategy not only enhances amino acid supply for milk synthesis but also contributes to reducing nitrogen losses and improving feed efficiency, making it a sustainable solution for modern dairy production systems.

Polysaccharides play a critical role in enhancing RUP by forming stable complexes with proteins, either through chemical interactions such as the Maillard reaction or by acting as physical barriers that slow down proteolysis in the rumen [32]. Among these, chitosan has been widely studied for its ability to increase RUP by creating polymer–protein complexes that reduce microbial access, thereby allowing a greater proportion of dietary protein to bypass ruminal degradation and reach the small intestine for enzymatic digestion [33]. This mechanism improves amino acid availability for absorption and utilization in highly productive ruminants and supports nitrogen pollution reduction quantified via RDP changes.

Kappa-carrageenan, a sulfated polysaccharide abundant in *E. cottonii*, exhibits similar protective properties. Its capacity to form ionic gel matrixes enables the development of encapsulation systems that resist microbial and enzymatic degradation under ruminal conditions [10]. Encapsulation using *E. cottonii* creates a controlled-release barrier, ensuring that proteins remain intact during ruminal fermentation and are subsequently digested in the abomasum and small intestine [34]. This structural integrity shifts the protein profile toward higher RUP, which is essential for meeting the amino acid requirements of high-yielding dairy cows, improving nitrogen utilization efficiency, and supporting milk protein synthesis.

The practical implications of this mechanism are significant. By reducing ruminal protein degradation and increasing bypass protein availability, *E. cottonii* enrichment contributes to improved feed efficiency and lactation performance. Furthermore, this approach mitigates nitrogen losses associated with excessive ruminal ammonia production, thereby reducing environmental nitrogen emissions. Compared with other encapsulating agents such as chitosan, *E. cottonii* offers advantages in terms of accessibility, cost-effectiveness, and ease of incorporation into feed formulations, particularly in regions where seaweed resources are abundant.

Overall, the data demonstrate that encapsulated *E. cottonii* can be strategically utilized to optimize rumen protein dynamics. This innovation represents a promising and sustainable approach for enhancing nutrient efficiency, improving milk yield, and supporting the economic viability of dairy farming systems.

### Gas production characteristics

This study demonstrates that enrichment with *E. cottonii* effectively reduces total gas and methane production during ruminal fermentation. As shown in Table 3, increasing levels of *E. cottonii* enrichment were associated with a marked decline in both total gas and methane output, with a strong correlation between enrichment level and methane reduction (R² = 0.87; Figure 3). Incorporating *E. cottonii* into GC represents a promising nutritional strategy, given that GC typically contain high levels of soluble protein, which can stimulate methanogenic bacteria by supplying nitrogen and amino acids utilized in methane synthesis [35].

Rich in bioactive compounds such as polysaccharides and antioxidants, *E. cottonii* enhances feed quality and fermentation dynamics. *In vitro* studies have demonstrated its capacity to mitigate CH_4_ emissions without impairing substrate degradability, primarily by modulating rumen microbial ecology and inhibiting methanogenic archaea—the key contributors to methane formation—thereby improving fermentation efficiency and supporting animal growth performance [36]. Furthermore, *E. cottonii* is a significant source of kappa-carrageenan, a polysaccharide known to improve digestibility and reduce total gas and methane production in ruminants [37].

These effects collectively enhance short-chain fatty acid synthesis and metabolizable energy availability, ultimately optimizing dairy cow productivity. This innovation has the potential to substantially reduce both ammonia and methane emissions, two key contributors to global warming. Its development represents a strategic effort to advance sustainable and environmentally responsible solutions. Moreover, this approach aligns directly with the aims of the 13th Sustainable Development Goal, which emphasizes the urgent need for climate action.

### Rumen performance characteristics

The analysis revealed that enrichment with *E. cottonii* resulted in the highest concentrations of total and individual VFAs compared to the control group without enrichment (Table 3), with a strong correlation (R² = 0.73; Figure 4). This finding is highly beneficial for livestock, as increased VFA production indicates greater nutrient utilization by the animal.

The observed increase in VFA concentrations can be attributed to the polysaccharide content of *E. cottonii*, which not only provides a protective effect on the rumen environment but also serves as an energy source that is metabolized into VFAs, including acetate, propionate, and butyrate [14]. Furthermore, enrichment up to 10% (GCC4) did not exert any adverse effects on rumen performance; on the contrary, it promoted VFA synthesis.

VFAs are generally classified into two categories: short-chain fatty acids, which represent a major energy source and can supply up to 80% of an animal’s daily energy requirements, including acetate, propionate, and butyrate; and branched-chain VFAs, such as valeric, isobutyric, and isovaleric acids. Branched-chain VFAs play a critical role in stimulating microbial protein synthesis and supporting the proliferation of cellulolytic bacteria, thereby enhancing fiber digestion and overall rumen function [38].

### *In vitro* digestibility of nutrients

*In vitro* digestibility evaluations of GC enriched with *E. cottonii* demonstrated significantly higher nutrient digestibility compared to the non-enriched control. Among the treatments, GCC4 exhibited the highest digestibility, which can be attributed to its *E. cottonii* inclusion that helps prevent premature nutrient degradation, thereby enhancing nutrient absorption in the intestine [14].

As shown in Table 3, increasing enrichment levels corresponded with improved *in vitro* digestibility, with strong correlations observed for IVCPD (R² = 0.94) and IVGED (R² = 0.92) (Figure 5). Enrichment with *E. cottonii* is considered an effective strategy for improving GC quality, as it can increase IVCPD by up to 88%. This effect is primarily due to its rumen-protective properties, which safeguard essential nutrients, particularly amino acids, from degradation in the rumen [38].

The mechanism of action is comparable to that of chitosan, offering notable nutritional benefits such as reduced ammonia nitrogen production and enhanced protein bypass to the lower gut [39]. Digestibility serves as a critical indicator of nutrient availability and FCE in ruminants; improved digestibility directly enhances nutrient intake, making it a key determinant of feed effectiveness [5].

### Milk yield and FCE

Cows receiving GC and GCC4 produced significantly higher milk yields and exhibited improved FCE compared to those on the control diet (CON). The highest milk yield was recorded in cows fed GCC4, reaching 10.05 kg/day (Table 4). In addition to the benefits of GC, enrichment with *E. cottonii* plays a pivotal role in enhancing milk yield.

This improvement is attributed to the ability of *E. cottonii* to increase feed digestibility and provide a rumen bypass effect for protein, as reflected by elevated RUP levels. Consequently, more nutrients become available for milk synthesis [40]. Similar to chitosan, kappa-carrageenan present in *E. cottonii* enhances amino acid absorption, which directly influences milk yield and serves as a precursor for prolactin, a key hormone in lactation [41].

Feed quality remains the primary determinant of milk yield in HF dairy cows. As shown in Table 3, GCC4 with 10% *E. cottonii* enrichment achieved the highest FCE, indicating superior feed efficiency. FCE is largely influenced by feed characteristics such as CP content and nutrient digestibility, which optimize milk output [42]. Thus, enriching GC with *E. cottonii* represents an effective strategy to improve feed quality, enhance milk yield, and maximize FCE, enabling higher milk yields from the same feed intake [32].

### Economic performance and feed affordability

Feed cost represents a major challenge for dairy farmers, as it accounts for approximately 70–80% of total production expenses in dairy farming. Key economic indicators such as feed efficiency, feed cost, milk yield, and milk price significantly influence farm profitability [43].

The inclusion of GC and GCC4 in the diet demonstrates strong potential for adoption by farmers, as these feeding strategies yield higher daily profits compared to the conventional diet (CON). Notably, GCC4 achieved the highest daily profit despite its slightly higher feed cost relative to GC, owing to its superior milk yield performance.

The approach employed in this study emphasizes the utilization of locally available feed resources with optimized nutrient composition and high digestibility through fortification with *E. cottonii* meal. This enrichment strategy is considered an innovative solution, as it enhances FCE, thereby reducing the effective cost per unit of milk produced and improving overall economic returns.

Compared with other RPA applications such as chitosan, the use of *E. cottonii* meal offers substantially greater cost efficiency. Chitosan is relatively expensive, ranging from 44.50 to 59.40 USD/kg, with an optimal inclusion rate of only 2% in dairy cow concentrate. In contrast, *E. cottonii* meal is far more economical, costing 3.55–4.45 USD/kg and allowing an optimal inclusion level of up to 10% in concentrates. This comparison indicates that adopting *E. cottonii* can reduce feed additive costs by approximately 60%–70% relative to chitosan, making it a more feasible and scalable option for dairy production systems, particularly in resource-limited or smallholder contexts.

### Milk quality

The primary components determining milk quality are fat and protein content, both of which are strongly influenced by feed quality. In this study, milk quality complied with the Indonesian National Standard for dairy products. Notably, cows fed GCC4 exhibited higher fat and protein concentrations in milk, indicating improved nutrient absorption and utilization for milk component synthesis [44].

Beyond fat and protein, the quality of whey and curd was also evaluated in Enrekang, where curd yield is of particular economic importance to farmers, as milk is traditionally processed into dangke (a local soft cheese) for sale. GCC4 produced the highest curd yield, attributable to its elevated solids content, including fat, protein, lactose, and SNF.

Milk protein and fat levels are critical predictors of curd yield, as specific thresholds are required to achieve desirable texture and consistency during curd formation. Conversely, whey and curd yields exhibit an inverse relationship; an increase in whey volume typically corresponds to reduced curd production, signaling lower milk solids content [20].

## CONCLUSION

The present study demonstrates that enrichment of GC with *E. cottonii* markedly improves feed quality, rumen protein utilization, and overall dairy cow performance. Increasing inclusion levels of *E. cottonii* significantly reduced RDP while enhancing RUP, indicating improved protein protection and nitrogen utilization efficiency. This was accompanied by significant improvements in *in vitro* digestibility parameters (IVDMD, IVOMD, IVCPD, and IVGED), reduced methane production and total gas output, and enhanced rumen fermentation characterized by increased concentrations of VFAs. In the *in vivo* phase, cows receiving GC enriched with *E. cottonii* (GCC4) exhibited higher milk yield, improved FCE, and superior milk quality, particularly in fat and protein content, along with increased curd yield. Economic analysis further revealed reduced feed costs and increased daily profit, highlighting the practical benefits of this feeding strategy.

From a practical perspective, the use of *E. cottonii* as a natural and cost-effective RPA offers a viable alternative to conventional protein protection technologies such as chitosan. Its ability to enhance nutrient utilization while simultaneously reducing methane emissions supports both productivity and environmental sustainability. The availability and relatively low cost of *E. cottonii* make this approach particularly suitable for smallholder dairy systems, where access to expensive feed additives is limited. Furthermore, the integration of nutritional, metabolic, and economic benefits positions this strategy as a promising tool for improving farm-level efficiency and profitability.

A major strength of this study lies in its integrated design combining *in vitro* and *in vivo* evaluations, allowing comprehensive assessment of nutritional mechanisms alongside production performance and economic outcomes. The inclusion of multiple fortification levels of *E. cottonii* provides valuable insights into dose-dependent responses, while the evaluation of rumen fermentation, digestibility, methane production, and milk quality ensures a holistic understanding of its effects.

However, certain limitations should be acknowledged. The study was conducted under specific environmental and management conditions, which may influence the generalizability of the findings. The sample size, although adequate for detecting treatment effects, remains relatively limited for broader population-level inference. In addition, long-term effects of *E. cottonii* supplementation on animal health, reproductive performance, and product quality were not evaluated. Variability in seaweed composition due to seasonal or geographic differences may also affect consistency of results.

Future research should focus on long-term feeding trials to evaluate the sustainability of responses and potential impacts on animal health and reproduction. Further investigation into the molecular and microbiological mechanisms underlying rumen modulation by *E. cottonii* is also warranted. Comparative studies across different production systems, breeds, and environmental conditions would help validate its applicability at a wider scale. Additionally, optimization of inclusion levels under field conditions and evaluation of processing methods to enhance bioavailability may further improve its efficacy.

In conclusion, enrichment of GC with *E. cottonii* represents an effective and sustainable nutritional strategy to enhance rumen protein utilization, improve digestibility, reduce methane emissions, and increase dairy productivity and profitability. This approach offers a practical and scalable solution for advancing efficient and environmentally responsible dairy production systems.

## DATA AVAILABILITY

The data generated during the study are included in the manuscript.

## AUTHORS’ CONTRIBUTIONS

RFU, AA, and ZR: Conceived, designed, and coordinated the study and drafted the manuscript. RFU and ZR: Conducted field sampling, data collection, laboratory work, and data entry. RFU, AA, MMR, JM, and KU: Performed statistical analysis. RFU, ZR, JM, and MMR: Interpreted statistical results. RFU and ZR: Conducted field experiments and tabulated data. All authors have read and approved the final version of the manuscript.
